# The role of husbands in maternal health and safe childbirth in rural Nepal: a qualitative study

**DOI:** 10.1186/s12884-015-0599-8

**Published:** 2015-08-04

**Authors:** Sarah Lewis, Andrew Lee, Padam Simkhada

**Affiliations:** Department of Geography and Section of Public Health, School of Health and Related Research, University of Sheffield, Sheffield, UK; Section of Public Health, School of Health and Related Research, University of Sheffield, Sheffield, UK

## Abstract

**Background:**

The role of husbands in maternal health is often overlooked by health programmes in developing countries and is an under-researched area of study globally. This study examines the role of husbands in maternity care and safe childbirth, their perceptions of the needs of women and children, the factors which influence or discourage their participation, and how women feel about male involvement around childbirth. It also identifies considerations that should be taken into account in the development of health education for husbands.

**Methods:**

This qualitative study was conducted in four rural hill villages in the Gorkha district of Nepal. Semi-structured, in-depth interviews were conducted with husbands (n = 17), wives (n = 15), mothers-in-law (n = 3), and health workers (n = 7) in Nepali through a translator. Interviews were transcribed and analysed using axial coding.

**Results:**

We found that, in rural Nepal, male involvement in maternal health and safe childbirth is complex and related to gradual and evolving changes in attitudes taking place. Traditional beliefs are upheld which influence male involvement, including the central role of women in the domain of pregnancy and childbirth that cannot be ignored. That said, husbands do have a role to play in maternity care. For example, they may be the only person available when a woman goes into labour. Considerable interest for the involvement of husbands was also expressed by both expectant mothers and fathers. However, it is important to recognise that the husbands’ role is shaped by many factors, including their availability, cultural beliefs, and traditions.

**Conclusions:**

This study shows that, although complex, expectant fathers do have an important role in maternal health and safe childbirth. Male involvement needs to be recognised and addressed in health education due to the potential benefits it may bring to both maternal and child health outcomes. This has important implications for health policy and practice, as there is a need for health systems and maternal health interventions to adapt in order to ensure the appropriate and effective inclusion of expectant fathers.

**Electronic supplementary material:**

The online version of this article (doi:10.1186/s12884-015-0599-8) contains supplementary material, which is available to authorized users.

## Background

Men’s roles in safeguarding maternal health have gained increased interest in recent years [1, 2]. Men can affect pregnancy and childbirth through responding to complications, seeking medical help, paying for transport, and allocating household resources [3, 4]. However, the role of husbands in maternal health is often overlooked and neglected.

In South Asian contexts, research has found that men possess little knowledge and experience regarding maternal health [5–7]. A lack of knowledge regarding complications and danger signs during pregnancy and delivery has been frustrating for husbands and has prevented their involvement [2]. This lack of knowledge undoubtedly affects maternal health outcomes. For example, Brunson’s (2010) study shows that despite the fact they may not be knowledgeable about birth, in emergencies men control the situation through their decision making [8].

Although traditionally a women’s role, studies have shown increasing desire of women for their husbands’ involvement, including during labour [8–10]. Mumtaz and Salway (2009) suggest that there is a need to move away from seeing men as “oppressors” and instead recognise them as partners in this realm [11]. Yet overall men’s involvement in maternal health is not well understood and has been little researched compared to other areas of sexual and reproductive health [6, 12, 13].

Since the 1994 Cairo International Conference on Population and Development (ICPD), which recognised that working with men is needed for effective change, male involvement in reproductive health issues has been well documented [14]. The effects of men and the sociocultural construction of masculinities on women’s reproductive health outcomes are also recognised [11]. Of research and interventions that have incorporated men these have been in the areas of family planning, HIV, and sexually transmitted diseases.

The effect of male involvement on the health of the newborn has also been recognised. In Alio et al’s (2010) study of feto-infant health they acknowledge that paternal behaviours in the maternal period can have long-lasting effects on the child’s health [15].

Nepal has one of the highest maternal mortality ratios in Asia, at 281 deaths per 100,000 live births [16, 17]. Multiple risk factors have been identified including the absence of skilled care at birth, delayed health-seeking and lack of access to health facilities [18]. These risk factors are prominent in rural areas and particularly relevant for Nepal as 90 % of the population resides in rural areas and nationally only a third of deliveries occur in health facilities [19, 20]. The majority of births occur at home and many women deliver with relatives, friends, untrained traditional birth attendants, or even alone, with its attendant risks [3, 21–23].

Currently, pregnancy and childbirth in Nepal are seen very much as a woman’s health issue [8]. It is traditional practice for the daughter-in-law to reside with the husband’s family after marriage and for decisions about maternal health care to be made by the mother-in-law [3, 24, 25]. It is also the practice for women to be assisted with members of the same sex during labour [26]. Consequently, maternal health programme interventions have looked at addressing issues of poor nutrition, lack of awareness, and health seeking behaviour predominantly through this gender lens.

However, given that many births in Nepal take place at home there is a need to look at care at the household level. In particular, as men’s knowledge and attitudes to health are significant factors influencing women’s maternal health [24], there is an urgent need to understand male perceptions of their role in maternal health and that husbands are educated and enabled to make timely decisions [7, 27]. More research is imperative into the types of care men may provide during pregnancy and childbirth [12].

This study explores the role of expectant fathers and assesses their need for health education about safe childbirth and maternal health in rural Nepal. Specifically, we sought to elucidate the nature of the husbands’ roles and involvement, the factors which influence or discourage their involvement, and their perceptions of the mother’s and the child’s needs. We also wanted to understand how women feel about male involvement in maternity care and identify considerations that should be taken into account in the development of health education for husbands.

## Methods

### Setting

The research took place in four villages in the Gorkha district, located in the Western region of Nepal. This rural area is extremely remote, with limited infrastructure including roads, communications, transport and equipped health facilities. The health facilities available are limited and varied in each village, with only one possessing a birthing centre, which is a three hour walk from most villages researched. Female Community Health Volunteers are present in each village.

The 2011 Census shows that the Gorkha district has a population of 271,061 [28]. The predominant religions followed are Hinduism (203,702 people), Buddhism (51,766) and Christianity (8,860). The main caste/ethnicity is Gurung (53,342), followed by Brahman – Hill (41,229), and Chhetree (31,479), among others [29].

### Study design, participants and sampling procedure

This study used a qualitative approach involving semi-structured, in-depth interviews with local people and health workers to explore feelings, understandings, and perceptions of male involvement.

Basic interview schedules were devised prior to research (see Additional file 1 for the original interview schedule). A local non-governmental organisation working in the area, PHASE Nepal, was consulted to ensure that the questions were suitable for the context. The interview schedule was also pre-tested with a Nepali translator for clarity.

Data collection took place in 2012. Interviews were conducted in Nepali through a translator. Respondents were invited to participate if they met the following inclusion criteria: they were a father, a mother, a mother-in-law, a Female Community Health Volunteer or health worker employed by the Government or PHASE Nepal. A sample size of 20 respondents was originally planned, although it was recognised that this number could increase or decrease depending on theoretical saturation (when new data does not give new insights but instead confirms previous theories) [30].

Respondents were identified and approached either through snowball sampling by previous participants, or door-to-door by the researchers. Potential respondents were given verbal information about the study (an information sheet was read out by the translator) and invited to participate with their willing consent. Consent was obtained and verified by thumbprint or handwritten signature before the start of the interviews. To ensure anonymity and confidentiality participants are not named in this study.

A total of 35 interviews were conducted with household respondents including 17 fathers, 15 mothers and three mothers-in-law. Seven key informant interviews were also conducted with PHASE Nepal health workers, Government health workers and Female Community Health Volunteers, based on their knowledge of maternal health in the study area (see Table 1). This larger sample size was achieved as the researchers wished to explore attitudes across each of the four villages in the study. After the 35 interviews with household respondents, theoretical saturation and informational redundancy was reached.

**Table 1:** Profile of respondents

Interview Number	Respondent
3	Husband 1
5	Husband 2
7	Husband 3
8	Husband 4
9	Husband 5
12	Husband 6
13	Husband 7
18	Husband 8
20	Husband 9
22	Husband 10
23	Husband 11
25	Husband 12
27	Husband 13
30	Husband 14
34	Husband 15
41	Husband 16
43	Husband 17
1	Wife 1
2	Wife 2
10	Wife 3
15	Wife 4
17	Wife 5
19	Wife 6
21	Wife 7
28	Wife 8
32	Wife 9
33	Wife 10
35	Wife 11
36	Wife 12
39	Wife 13
40	Wife 14
42	Wife 15
16	Mother-in Law 1
24	Mother-in-Law 2
37	Mother-in-Law 3
4	Health worker 1
6	Health worker 2 (Female Community Health Volunteer)
11	Health worker 3
26	Health worker 4
29	Health worker 5 (Female Community Health Volunteer)
31	Health worker 6 (Female Community Health Volunteer)
38	Health worker 7

Where permission was granted by the respondents, in-depth interviews were tape-recorded and supplemented with note-taking. Where respondents were uncomfortable with the tape recorder, notes were taken. Interviews were conducted in the mornings and evenings, before and after respondents went to work and were conducted in the home. Key informant interviews were conducted in the work place. Health facilities were also visited in all of the villages and at the nearest town.

### Data analysis

Half of the interviews were transcribed by hand in the field, enabling any unclear issues to be clarified by the translator. Preliminary analysis of the transcribed interviews in the field also enabled the interview schedule to be iteratively modified so that areas of interest could be explored in more detail in subsequent interviews. The remaining interviews were transcribed upon return from the field by computer. Open coding was undertaken by hand and a grounded theory approach was taken to data analysis, enabling theory to emerge from the data and from the experiences of participants [31, 32]. Transcripts were read for familiarity and notes made. These notes were then formalised into codes. Codes were categorised and axial coding was undertaken to tease out aspects and properties of each main code.

### Ethical considerations

The study protocol was approved by the University of Sheffield School of Health and Related Research’s Research Ethics Committee.

## Results

**Fig. 1:**
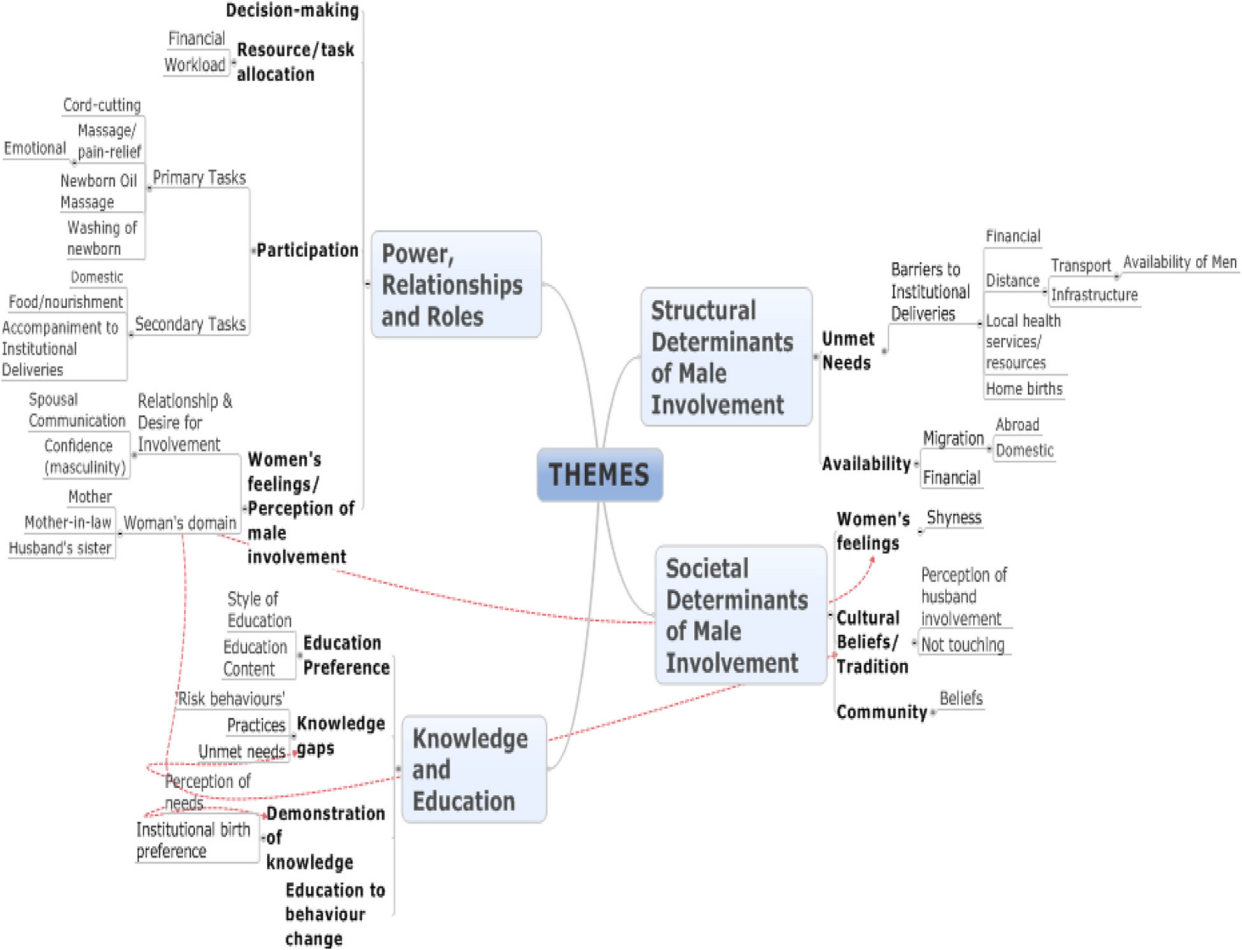
Mind map of themes, sub-themes and sub-sub themes derived from analysis

During the analysis, codes which related to each other were grouped together to form four main themes, sub-themes and sub-sub themes, as demonstrated in Fig. 1.

### Structural determinants of male involvement

#### Barriers to institutional births

There are multiple structural barriers to women delivering in health facilities. For example, the facilities at existing health posts were limited and only one village possessed a birthing centre. This meant pregnant women had to travel long distances on difficult and potentially dangerous paths to reach an equipped health facility:

“At the time of delivery the woman should be carried. But in May, June and July there will be heavy rainfall and the road will be slippery, so it is very difficult to go from here to there”. Husband 8

There are also financial barriers such as the cost of transporting the women to the health facilities. Even when health facilities are available, not infrequently, they would not be staffed.

#### Availability

The husbands’ availability is another reported determinant that restricts male involvement in maternal health and childbirth as this is particularly associated with labour migration trends. As one woman reported:

“To earn financially the husband has to go abroad … (this) stops men being involved”. Wife 8

Their absence restricts the role that they play in their wife’s pregnancy and childbirth, and increases the significance of the role played by others, such as the mother or mother-in-law, at the time of delivery:

“The trend is for the husband being there, but (many) husbands go away to work so the mother-in-law stays here”. Health worker 2 – Female Community Health Volunteer

In some cases, when the husband was present, other women or health workers were often not available and therefore the husband’s presence was necessary as they were the only person available to assist with the delivery.

### Non-structural and cultural determinants

#### Cultural beliefs (including postpartum seclusion)

Cultural beliefs could also restrict male involvement. These include the idea that birthing difficulties would be encountered if the husband was present during childbirth. Another tradition followed is postpartum seclusion, where physical contact has to be avoided with the postnatal woman for between three and seven days after delivery. This also includes restrictions on food preparation. The degree to which this tradition was followed varied between households.

“The culture stops men being involved; the culture is not to touch the woman (who has just delivered), not only the husband but the mother-in-law and father-in-law too. No one should touch the woman for up to seven days”… Health worker 1

#### Women’s feelings

We found a range of preferences for male involvement. Women commonly reported feeling embarrassed with having their husbands present during childbirth. We also found that some women were reluctant to share details of pregnancy and childbirth with their husbands. As one health worker explained:

“Most of the women feel ‘shy’ with their husband; if he is absent then they will be fine. Mostly they don’t want their husband to be there.” Health worker 1

Paradoxically, depending on the quality of the relationship between husband and wife, some women wanted their husband’s involvement despite childbirth being seen largely as a female domain. We found instances when some women were uncomfortable with having their mother-in-law present and preferred their husband’s presence instead:

“I wanted my husband to be there… (I) feel shy with other people but not my husband”. Wife 14

Interestingly, we found that the majority of women interviewed actually expressed a desire for their husbands to be involved with their maternal health, running counter to the idea that women do not want their husbands to be present. Some women felt ‘closeness’ to their husbands and were more able to share their feelings around pregnancy and childbirth. Some women felt safer and more secure having their husbands present:

“The husband will help and in every problem there will be a solution if the husband will be there.” Wife 8

#### Women’s domain

Pregnancy, childbirth and care of the newborn were largely considered as activities to be conducted by the woman herself or by other women, particularly the mother-in-law, the wife’s mother and the husband’s sister. This gendered perception significantly influences the degree of the husband’s involvement:

“(She) doesn’t want him to be there because her husband does not do anything. She cooks the food, and everything else… Her husband does not help out, she has to do the work herself, cut the cord, care for the baby”. Wife 2

“Difficult for husband to say to pregnant and delivery woman but very easy for the mother-in-law to give women advice like go there for antenatal check-up. Easy to (talk about) complications, share between women and women… easy for mother-in-law to talk with women rather than men”. Husband 6

When both the husband and other women were present, the typical gendered division of labour was adhered to. For example, the mother-in-law was responsible for taking direct care of the woman, whilst the man’s role was largely secondary, such as to prepare food, provide financially and undertake minor tasks such as burying the umbilical cord.

#### Gendered roles

We found that overall husbands do not participate in the hands-on delivery. Instead, when they are involved, their roles are largely secondary and supportive, and most notably had to do with providing and preparing food. As one husband describes:

“(I) only cooked food and gave. Didn’t do anything beside that”. Husband 5

This is partly due to the women’s restricted access to the kitchen during the postpartum seclusion period and as such may be perceived to be a necessity. The other women, such as the mother-in-law, instead had the main role of assisting directly with the childbirth:

“Mostly if husbands help it is for the outer work like helping (with) the food, to collect the food, whom to call during the delivery… The husband works in that area but women sit there at childbirth”. Health worker 4

Some of the primary tasks in the postnatal period that may be undertaken by the husbands included the cutting of the umbilical cord or providing the necessary instruments to do so. This depended on whether this was perceived to be a woman’s role and who else was present. However, in many cases the woman herself cuts the cord:

“I boiled the water and gave (it to her) but I did not touch the woman. She herself cut the cord”. Husband 4

Other secondary tasks commonly undertaken by the men included massaging their wife during labour and washing the newborn afterwards. Of note, although some men described themselves as being present at the time of delivery, whilst they were physically present they tended not to actually participate in the delivery.

Men also accompanied their wives to health facilities, due to the need to be stretchered, to provide financially, and as they perceived it to be an obligation as a husband. However, men were then expected to wait outside the delivery room. That said, at one health centre in a nearby town husbands were now being encouraged to enter the delivery room; as a health worker explained that witnessing the pain of childbirth could facilitate future family planning.

We also found that decision making varied between households, being undertaken by both husbands and the mother-in-law. Traditionally, the mother-in-law occupies the top position in the household:

…“mother-in-law is the main person in the household officially, so we have to be in order”. Husband 10

However, decision making is increasingly no longer the exclusive domain of the mother-in-law:

“Previously there is a trend of (decisions being made) by the mother-in-law. Now the decisions (are made) by the husband”. Husband 8

Although their decision making is dependent on their availability, husbands did make decisions in relation to seeking medical help, determining the place of birth, allocation of family resources including finances, and task allocation such as with regards to the domestic workload. In some cases, particularly in the absence of their husbands, the women made these decisions independently.

### Knowledge and education

#### Gaps in knowledge

We found that although some husbands were knowledgeable about aspects of pregnancy and childbirth, there were significant gaps. Many husbands were aware of the benefits of delivering in a health facility and stated that they would prefer their wives to have an institutional delivery attended by a trained health worker. Interviewees also demonstrated some awareness of potential obstetric complications such as obstructed labour (for example, “if the baby cannot come out” Husband 12). However, overall the husbands’ knowledge of the range of danger signs in pregnancy, during delivery, and in the neonatal and postnatal periods was lacking.

We found that there is an increased awareness of the importance of cleanliness. For example, references were made to “cutting the cord with a new, sterilised blade”. However, health workers reported that old blades, including those used for shaving, were still being used. Traditional practices such as the application of ash or turmeric oil to the umbilical cord were also common and the majority of respondents also reported washing the newborn immediately after delivery.

Husbands did seem aware of the need to intervene to assist their wives as opposed to practicing the culture of “not touching” and also acknowledged the importance of nutritious food and of making birth preparations. Some acknowledged the importance of reducing the women’s workload in pregnancy although this may not be practicable in view of the harsh living conditions in the remote areas. Somewhat surprisingly, husbands were aware of their lack of knowledge and voiced a sense of helplessness in knowing how to care for their wives:

“This is the problem - we don’t know what to do, what food to give during pregnancy and after childbirth, how to take care”. Husband 12

#### Desire for education

The majority of respondents, including wives who did not want their husbands to be present, thought that educating husbands would be beneficial for the mother and child’s health. A desire for further education was voiced:

“It’s necessary to teach the men… if a complication occurs and the husband is taught then they will (know to) take the wife to hospital”. Wife 11

“(I) want to learn more about maternity and childcare: what should be done during pregnancy (for the) woman, what should be done after childbirth to make the baby healthy”. Husband 2

The perceived benefits that education would bring included: husbands knowing more about women’s health, a reduction in the women’s workload, positive decision making and improved relationships.

#### Future education

Preferences over the style of health education varied. When education of husbands was desired there was a preference for education to be delivered in a group setting. However, due to women’s shyness in the presence of men, one-to-one education was preferred by some. The use of picture cards, flipcharts, drama, singing and dancing was thought to be effective. That said the feasibility of such community-based education may be limited due to the men’s absence.

## Discussion

Previous studies have highlighted significant barriers to male participation as pregnancy and childbirth are often perceived as a woman’s realm [33, 34]. Men’s involvement is culturally discouraged as many of the important barriers for husbands, like social pressure, lack of knowledge and spousal communication are gender related [34]. Pregnancy and childbirth are particularly perceived as gendered processes and there is consequently considerable social stigma that leads to male shyness and embarrassment with regards to pregnancy-related discussions between husbands and wives [2, 35]. There is however a gradual shift towards improving male involvement in maternal health. As noted by Sternberg and Hubley “although perhaps no longer seen as part of the problem, men have yet to be seen as part of the solution” [36].

One of the surprising findings of this study was the positive desire by both Nepali husbands and wives for there to be greater male involvement. This probably reflects changing social attitudes in Nepal and contradicts arguments which stress the persistent centrality of women in pregnancy and childbirth that relate to differing gender ideologies. One of the possible explanations for this phenomenon is that male migration to urban centres in Nepal and abroad has increased exposure to foreign ideologies that influence gender norms [2]. Changing attitudes towards traditional norms is also recognised in Brunson’s (2010) study, where although historically there was a lack of male involvement, women are now more vocal about wanting support in birth from their husbands [8].

That said, the openness to have male involvement in pregnancy and childbirth is not universal and there is a spectrum of involvement desired. At one end of the spectrum traditional beliefs persist and influence pregnancy and childbirth as a woman’s domain, where women only desire female presence. At the opposite end of the spectrum women desire their husband’s involvement and husbands are able to act on this, being both present and undertaking tasks such as cord-cutting, although still not actually participating in the hands-on delivery.

The findings revealed that males’ roles are complex. Although men do not participate in the hands-on delivery, by being present they acquire multidimensional roles [24]. Their roles are largely secondary in nature and particularly relate to food provision. There was also some involvement in primary tasks such as umbilical cord cutting, which suggests that traditional gender roles are less concrete. Husbands also have an important role in decision-making especially with regards to how limited family resources are utilised, although a hierarchical system does still exist whereby husbands and mothers-in-law are predominant decision makers [37]. In this way their roles can have a direct effect on women’s maternal health, and the health of the newborn.

We have also identified factors that discourage husband involvement such as the prevailing traditional beliefs, culturally-determined gender roles around pregnancy and childbirth with the mother-in-law upholding an important role, discouragement by the medical system for men to enter the delivery room and women’s shyness, especially during delivery, of involving their partners. However, rather than deliver alone, women prefer to have their husbands present if available. We also found a paradoxical desire; the existing husband-wife relationship did influence male involvement, consistent with Carter’s (2002) findings:

‘The centrality of (romantic) love to these participants’ understanding of male involvement is one indication that the involvement of husbands is qualitatively different from that of other family members or friends… many women and men suggested that receiving social support (advice, money, accompaniment, encouragement) from mothers or mothers-in-law is not the same and often not as good as that from husbands’ [38].

We found that stigma did not emerge to a great extent, with many men not hesitant to discuss their involvement, which is in contrast to many existing studies, such as Mumtaz and Salway’s (2009) study in Pakistan, where in joint families a man is deemed ‘shameless’ if he displays too great an interest in his wife’s pregnancy [11].

Numerous studies and health initiatives have been set up to improve maternal health in developing countries such as Nepal. However, most of these initiatives have tended to solely focus on the women. Whilst it is clear that pregnancy and childbirth are culturally gendered roles, as supported by Hoga et al’s (2001) study in Brazil, we argue that this does not mean that there is no male influence in the birth process [39].

Maternal health initiatives that focus solely on women may be flawed as they ignore the social context that they live in. These contextual factors are particularly important in patriarchal societies in South Asia where women have low social status and may be dependent on their husbands with regards to decision making [7, 40–42]. For example, one of the key roles played by husbands is as economic providers which are reported to result in women’s dependence on issues such as food and nutritional intake [12, 24]. In this economic provider role, husbands act as potential gatekeepers to women’s healthcare-seeking. Their perceptions of maternal needs are of crucial importance as they can determine delays in health seeking.

There is also a clearly expressed desire by both men and women for health education. Some key health education topics to be considered include the prompt recognition of maternal and newborn danger signs. It is important to note, however, that whichever health education initiatives are implemented, they will need to be sensitive to the range of responses and attitudes of men and women to male involvement. Whilst we have argued that there is a case for increasing male involvement in pregnancy and childbirth, it would be wrong to assume that there is a universal desire for this. Male involvement may be the norm in the West [41] but in other countries women may resent their husband’s involvement in delivery or even presence in the delivery room [43–45]. Rather than assume the desire for male involvement is universal, interventions must therefore consider ideas, fears, preconceptions and if, and in what ways, women wish for male involvement in their maternal health [2].

It has been reported elsewhere that greater participation by husbands in maternal health has led to increased use of antenatal care, institutional deliveries, improved spousal relationships and women’s desires for male involvement [46]. Similarly, in rural Guatemala, Carter (2002) found that husbands often participated through providing money for antenatal care, giving advice during pregnancy and the postpartum period, and ‘accompanying’ wives or waiting in a nearby room during home births [38]. In urban Nepal, Sapkota et al. (2012) found that husbands positively supported their wives during childbirth and demonstrated a willingness to do so [47]. The husbands’ presence at childbirth has also been found to have positive long-term effects. For example, witnessing the birth has been cited as educational for husbands about the process, which translated into more respect for women generally, and smaller family sizes [47].

This study further adds to these findings and stresses that it is important to involve husbands. In rural Nepal, husbands may be the only person available when a woman goes into labour and in the absence of skilled health providers, equipped health facilities, or other family members their secondary roles may also have a direct effect on the health of mother and newborn [45].

Previous studies have examined ways to encourage male involvement and to disseminate information effectively [14]. In order to educate effectively multiple considerations must be taken into account to ensure interventions are tailored to the specific needs of husbands, wives and the community [14]. These include identifying gaps in knowledge prior to interventions, considering how men would prefer to be educated, whether women would in fact be in favour of this, and ensuring that the content is realistic for the local situation considering the health facilities and services available [7, 14]. In particular, the spectrum of how far male involvement is desired is a significant consideration in the development of health education and there is a need to be sensitive to the range of responses and attitudes [2].

As some women in the community have traditional values and experience shyness when discussing maternal health matters in the presence of men, we suggest that group education be separated by gender with additional options for follow-up couple and individual counselling where desired, thereby ensuring education is culturally appropriate and considerate of varying attitudes [14]. With regards to the content of education we argue that expectant fathers should be educated on prompt recognition of maternal and newborn danger signs as they may be the only person present at this time [48]. This is especially important where health facilities are a considerable distance away, and given men’s roles as decision makers and gatekeepers to women’s healthcare. When addressing cultural traditions such as postpartum seclusion, this must be handled sensitively [49].

We found that the communities researched are undergoing a gradual transition in gender norms, where traditions are upheld in some households but evolving in others. There is a spectrum of acceptability of male involvement, where at one end pregnancy and childbirth are female domains, and at the other end the presence of husbands is desired. As a result, the husbands’ roles are complex and sometimes contested. These roles are both influenced and discouraged by factors such as availability, traditional beliefs such as the predominant role of women, and gender roles.

### Limitations

The number of men available for interview was reduced due to labour migration to urban areas or abroad. Men that we could interview were those who had not migrated away and had therefore been present for the birth of at least one child or had accompanied his wife to a health facility. To address this, we purposively sought to interview women whose husbands had not been present at any birth, to explore the reasons for this and their feelings around this.

One other potential limitation of this study was that traditional birth attendants were not interviewed. Given their role, it would be useful for future research to explore how they may affect male involvement in maternal and child health practices.

Finally, whilst the researcher was female, it was anticipated that the male gender of the translator could be a limitation when talking to women. Ideally we would have preferred using a female translator but this was not possible for practical reasons.

## Conclusions

Nepal is a diverse country that is undergoing considerable demographic change. Significant variations exist both between and within communities due to differences of socio-economic status, ethnicity, religious beliefs, literacy and caste. Further research is needed to explore how these variations affect male involvement in maternal and child health practices, particularly between rural and urban communities. It will also be important for future research to investigate the longer-term effects of male education interventions, and male involvement during pregnancy and childbirth itself, on maternal health and safe childbirth outcomes.

Men have a significant role in safe childbirth and maternal health even through their secondary roles. Recognising the value of their roles is a key step towards no longer seeing men on the periphery but as part of the solution [36]. There is a need to include men in maternal health promotion and education, to increase both their knowledge and participation in the birth process, whilst recognising that there are various important considerations to take into account. We conclude that greater male involvement has the potential to deliver considerable benefits for maternal and child health.

## Additional file

Additional file 1:
**Basic Interview Schedule.** Basic interview schedule, devised prior to data collection. (PDF 211 kb)
