# Supplementary material for: The role of husbands in maternal health and safe childbirth in rural Nepal: a qualitative study
Source: BMC Pregnancy Childbirth. 2015 Aug 4;15:162. doi: 10.1186/s12884-015-0599-8 (PMC4523911; doi:10.1186/s12884-015-0599-8)
Supplement: Additional file 1: — Basic Interview Schedule. Basic interview schedule, devised prior to data collection. (PDF 211 kb) [file 12884_2015_599_MOESM1_ESM.pdf]

Additional File 1: Basic Interview Schedule

Household Respondents

Fathers and mothers

After introductions, ask each respondent:

- How old are you?
- Who lives in your house?
- How many children do you have? How old are they?
- How many pregnancies have you/ your wife had?
- Have you/ your wife/ your husband been to school?

Main interview questions

- Where did you/ your wife/ daughter-in-law give birth? (*inside home, outside home, at health facility)* Can you tell me why you gave birth there? (*to explore whether services are available/ accessible)*
- Was there anywhere else you would have preferred to give birth? Are you available to access these services?
- What do you think women and the child's needs are during pregnancy, childbirth and after the baby is born? *(to explore knowledge of danger signs, safe cutting of the cord, care of the newborn)*
- What concerns you about your wife's pregnancy and childbirth? (*to explore fears, preconceptions, awareness of risks/danger signs)*
- Do you think anyone should be there at birth, and if so who/why?
- Can you tell me about what people in your culture think the father's role is in pregnancy, childbirth and after the baby is born? *(to explore how common it is for fathers to be present, if there is any stigma surrounding involvement)*
- Do you have any experience of childbirth, if so can you describe your role and experience of being there?/ Can you describe your experience of pregnancy and childbirth?
    - Can you describe your/his/other person's role in being there? *(to explore emotional/traditional as well as practical actions)*
    - What are the reasons this person was there?
    - Were there any positive effects of you/him being there?
    - Were there any negative effects of you/him being there?
    - Were there any complications? If so can you describe what happened?
- Did you want/want your husband to be there?
- Is there anyone who was not present at the birth that you want to be there?
- Did you/your husband being there have any effect on how much you/he could work or earn?
- Were any preparations made for the birth? *(to explore whether money is set aside for emergencies, safe delivery kits are purchased/used, emergency plan made)*
- Who makes the decisions about your wife's/ your care during pregnancy and childbirth?

- Have you (/your husband) had any education about how to look after your wife during pregnancy or childbirth? Do you feel there is more you would like (/like your husband) to be taught about? How would you want this to happen?

Mothers-in-law/ grandmothers

- Can you describe who makes decisions about an expectant mother's health in your culture?
- Do you think anyone should help the mother when she gives birth? If so who?
- Can you tell me about what you think your role is in your daughter-in-law's pregnancy and around the time of delivery?
- Can you tell me about what you think father's roles are in pregnancy/childbirth/ care of the mother and child after birth?
- Who was there at the birth of your grandchild? Can you describe what their role was?
- Can you tell me about how you feel about educating fathers about how to look after the wife when she's pregnant and how to deliver the baby?

Health workers

- Can you describe your role please?
- How long have you worked here?
- Who do you think is most involved in women's pregnancy in this area?
- Is it common for fathers to be present at the birth either at home or at health facility? Are there certain groups of people for which it is more common that the father would be there?
- If fathers are present, what stops other family members or health workers people being at the birth?
- Can you describe what you understand the role of men in maternity care and childbirth to be in the area please?
- What do you think the community's attitude to fathers being involved in maternity care and childbirth is like in the area? *(explore women's attitudes to male involvement)*
- Can you tell me what you think would make the biggest improvement to women's maternal health in this area?
- To your knowledge, can you describe what you believe fathers know about safe childbirth and maternity care?
- What are your opinions about educating fathers about safe childbirth and maternal health?
- How do you think educating men would be effective? (*Pictures, song/dance, individually, as a group?)*
- Are there currently any education services available to men?
